# Prevalence and Factors Associated with Prehypertension and Hypertension Among Adults: Baseline Findings of PURE Malaysia Cohort Study

**DOI:** 10.1016/j.ajmo.2023.100049

**Published:** 2023-06-23

**Authors:** Rosnah Ismail, Noor Hassim Ismail, Zaleha Md Isa, Azmi Mohd Tamil, Mohd Hasni Ja'afar, Nafiza Mat Nasir, Suraya Abdul-Razak, Najihah Zainol Abidin, Nurul Hafiza Ab Razak, Philip Joseph, Khairul Hazdi Yusof

**Affiliations:** a Department of Public Health Medicine, Faculty of Medicine, Universiti Kebangsaan Malaysia, Cheras, Kuala Lumpur, Malaysia; b Department of Primary Care Medicine, Faculty of Medicine Universiti Teknologi MARA (UiTM), Selayang Campus, Selangor, Malaysia; c Cardio Vascular and Lungs Research Institute (CaVaLRI), Pusat Perubatan UiTM, Kampus Sungai Buloh, Sungai Buloh, Selangor, Malaysia; d Institute of Pathology, Laboratory and Forensic Medicine (I-PPerForM), Universiti Teknologi MARA, Sungai Buloh, Selangor, Malaysia; e Department of Diagnostic and Allied Health Science, Faculty of Health and Life Sciences, Management and Science University, Shah Alam, Selangor, Malaysia; f Population Health Research Institute, Hamilton Health Sciences and McMaster University, Hamilton, ON, Canada

**Keywords:** Health screening, High blood pressure, Lifestyle modification, Obesity, Prehypertension

## Abstract

**Background:**

Although prehypertension and hypertension can be detected at the primary healthcare level and low-cost treatments can effectively control its complications, hypertension is still the world's leading preventable risk factor. Therefore, the present study aimed to determine its prevalence and its risk factors among Malaysian adults.

**Methods:**

A cross-sectional study involving 7585 adults was performed covering the rural and urban areas. Respondents with systolic blood pressure (SBP) of 120-139 mmHg and/or diastolic blood pressure (DBP) of 80-89 mmHg were categorized as prehypertensive, and hypertensive categorization was used for respondents with an SBP of ≥140 mmHg and/or DBP of ≥90 mmHg.

**Results:**

Respondents reported to have prehypertension and hypertension were 40.7% and 38.0%, respectively. Those residing in a rural area, older age, male, family history of hypertension, and overweight or obese were associated with higher odds of prehypertension and hypertension. Unique to hypertension, the factors included low educational level (AOR: 1.349; 95% CI: 1.146, 1.588), unemployment (1.350; 1.16, 1.572), comorbidity of diabetes (1.474; 1.178, 1.844), and inadequate fruit consumption (1.253; 1.094, 1.436).

**Conclusions:**

As the prehypertensive state may affect the prevalence of hypertension, proactive strategies are needed to increase early detection of the disease among specific group of those residing in a rural area, older age, male, family history of hypertension, and overweight or obese.

## 1 Introduction

Hypertension is a widespread global public health issue. It is also recognized as the leading modifiable risk factor for cardiovascular diseases (CVDs), with significant annual economic and health costs.^1^, ^2^, ^3^, ^4^ The World Health Organization (WHO) reports that 1 in every 4 men and 1 in every 5 women are diagnosed with hypertension, affecting nearly 1.13 billion people worldwide.^5^ The growing prevalence of hypertension is observed in countries of all income levels, with two-thirds of those affected living in low- and middle-income countries.

In Malaysia, the prevalence of hypertension as reported by the latest National Health and Morbidity Survey (NHMS) in 2019 was 30.0%, a slight reduction from the previously reported NHMS 2015 (30.3%) and NHMS 2011 (32.6%) results.^6^, ^7^, ^8^ Although the prevalence appears to be decreasing, nearly half of the cases were those with unknown hypertension. Without early detection, healthy lifestyle modifications, including diet and physical activity changes, cannot be implemented for those with prehypertension and unknown hypertension, thus increasing the probability of the former group progressing to hypertension and the latter group facing hypertension-related complications.

Current evidence indicates that hypertension is a multifactorial condition influenced by many risk factors, including genetic, sociodemographic, and behavioral factors.^9^^,^^10^ Age, ethnicity, and gender are among the nonmodifiable risk factors, while physical inactivity and unbalanced dietary choices leading to weight gain are often modifiable. Socioeconomic status and education have also been mentioned in multiple studies, as these two factors play a role in determining access to healthcare systems and awareness of healthy lifestyles.^11^, ^12^, ^13^

Little is known about which factors need to be prioritized for more tectonic intervention to reduce prehypertension and hypertension among Malay-dominant adults in Malaysia. Furthermore, studies focusing on factors of prehypertension were still limited in Malaysia. Therefore, this study aimed to fill this gap and to the best of our knowledge, this is the first attempt to study both factors of prehypertension and hypertension simultaneously involving large study samples from both rural and urban populations of Malaysia. The objective of this study is to determine the prevalence and factors associated with prehypertension and hypertension among adults residing in rural and urban areas of Malaysia.

## 2 Methodology

### 2.1 Study Design and Data Collection

This study was a regional substudy of participants from Malaysia enrolled in the multinational Prospective Urban Rural Epidemiology (PURE) study. A key objective of PURE is to understand the predominant health determinants for the development of noncommunicable diseases. PURE is an ongoing prospective cohort study over next 13 years; however, only the baseline data was included in this study. The comprehensive methodology of the overall PURE study has been explained in detail in previous studies.^14^^,^^15^ This community-based study included adults aged 35-70 years old.

In the Malaysia cohort, respondents were conveniently recruited from select urban and rural regions with the assistance of local community leaders. Health screening and health promotion booths were set up in the communities’ assembly halls, where interested and eligible participants were informed of the study. After agreeing to participate and providing written consent, their medical history was obtained and a basic physical examination was conducted. Home visits were also set to recruit other individuals living in the same household. Only individuals who intend to continue living in their current home for a further at least 4 years were selected. All data were obtained through face-to-face interview sessions by well-trained research assistants using a standardized and verified set of questionnaires.

This study was conducted according to the guidelines laid down in the Declaration of Helsinki and all the protocol of this study was approved by the Hamilton Health Sciences Research Ethics Board (PHRI; grant no. 101414). Local ethics approval was obtained from the Research and Ethics Committee of Universiti Kebangsaan Malaysia (UKM) Medical Center (project code: PHUM-2012-01) and the Research Ethics Committee of Universiti Teknologi Mara (UiTM) (REC/UITM/2007(10)).

### 2.2 Measures

The basic questionnaire consisted of 30 questions covering information on socioeconomic characteristics and baseline cardiometabolic risk factors. There were 20 questions on socioeconomic characteristics ranging from participants’ age and education level to employment status, whereas 10 questions were on the cardiometabolic risk factors. All participants were asked whether they had a medical diagnosis of other comorbidities such as diabetes and whether there have any family members with hypertension. As part of the questionnaire, health-related behaviors such as physical activity level was captured using the International Physical Activity Questionnaire (IPAQ) and fruit intakes were asked through validated food frequency questionnaire (FFQ). Body mass index (BMI) was calculated by dividing weight (in kilograms) by the square of height (in meters). Overweight was defined as a BMI greater or equal to 25 kg/m^2^ and less than 30 kg/m^2^, and obesity was defined as a BMI ≥30 kg/m^2^.

The questionnaire was developed by the Population Health Research Institute (PHRI) and later revised and validated by the Malaysian team of researchers to ensure its suitability with the local population. Face and content validity were carried out by the research team who are experts on public health–related studies.

Out of 15,378 respondents who participated in the study, only 10,031 (65.2%) individuals had given consent to answer FFQ. Of the 10,031 participants, only 7585 (75.6%) of them provided the completed questionnaire of sociodemographics such as comorbidities and family history of hypertension. Thus, the remaining 2446 respondents were excluded due to incomplete questionnaire answered.

### 2.3 Measurement of Blood Pressure and Definition of Prehypertension and Hypertension

Blood pressure was measured using a calibrated Omron automatic digital monitor (Omron HEM-757; Omron Corp, Tokyo, Japan) by a trained research assistant after 15 minutes of rest while the participants were in a seated position. The main hypertension definition used in this article was individuals who reported having hypertension and receiving blood pressure–lowering treatment or have had an average systolic blood pressure (SBP) of at least 140 mmHg, an average diastolic blood pressure (DBP) of at least 90 mmHg (prehypertensive), or both an SBP and DBP that exceeded the previously shown levels. Respondents with SBP of at least 140 mmHg and DBP of at least 90 mmHg (or both, as previously shown) were categorized as hypertensive.^11^ Two readings of SBP and DBP were taken at 5-minute intervals with appropriately sized cuffs based on a standard protocol. The average of the 2 readings was recorded and categorized as normal, prehypertension, or hypertension according to the Malaysian Clinical Practice Guidelines (CPG) on Hypertension 2018.^16^

### 2.4 Statistical Analysis

The data were analyzed using the SPSS version 26. The general characteristics of respondents were descriptively analyzed and presented as the numbers (and corresponding percentages). Multinomial logistic regression analysis was performed to investigate the potential determinants of the prehypertension and hypertension. Odds ratios (ORs) and 95% confidence intervals (CIs) were calculated. Statistical significance was set at *P* < 0.05. The model was adjusted for age, gender, education level, location, employment status, BMI, family history of diabetes and hypertension, and fruit consumption.

## 3 Results

Among the 7585 adults included in the study, majority were female (57%), who were on average aged 52 years (mean ± SD; 51.7 ± 9.5) and more than half received at least secondary level education (56%). In terms of location, about 62% resided in rural area as opposed to 38% in urban. The prevalence of prehypertension and hypertension were 40.7% and 38.0%, respectively (Table 1). Analysis of the factors associated with prehypertension and hypertension is shown in Table 2. Respondents aged 50 years and above had nearly 2 times the odds (adjusted OR: 1.723; 95% CI: 1.494, 1.987) of having prehypertension than those below 50 years of age. Males were more likely to have prehypertension (OR: 1.706; 95% CI: 1.478, 1.969) than females, while those residing in rural areas were twice as likely (OR: 1.694; 95% CI: 1.475, 1.946) to have prehypertension than those residing in urban areas. Participants who were overweight and obese were 2 (OR: 2.002; 95% CI: 1.739, 2.305) and 3 times (OR: 3.052; 95% CI: 2.53, 3.68) more likely to have prehypertension, respectively. Respondents who reported having at least one family member with hypertension were 1.4 times (OR: 1.391; 95% CI: 1.21, 1.598) more likely to have prehypertension than those without a family history of hypertension.

**Table 1:** General Characteristics of Participants (*N* = 7585)

Characteristics	Total	Frequency (%)		
		Normal	Prehypertension	Hypertension
	*N* (%)	1618 (21.3)	3085 (40.7)	2882 (38.0)
Age (year)				
≥50	4260 (56.2)	599 (14.1)	1544 (36.2)	2117 (49.7)
<50	3325 (43.8)	1019 (30.6)	1541 (46.3)	765 (23)
Gender				
Male	3259 (43)	550 (16.9)	1383 (42.4)	1326 (40.7)
Female	4326 (57)	1068 (24.7)	1702 (39.3)	1556 (36.0)
Education level				
Low	3378 (44.5)	560 (16.6)	1218 (36.1)	1600 (47.4)
High	4207 (55.5)	1058 (25.1)	1867 (44.4)	1282 (30.5)
Location				
Rural	4706 (62.0)	841 (17.9)	1910 (40.6)	1955 (41.5)
Urban	2879 (38.0)	777 (27.0)	1175 (40.8)	927 (32.2)
Employment status				
No	4045 (53.4)	798 (19.7)	1522 (37.6)	1725 (42.6)
Yes	3523 (46.6)	813 (23.1)	1558 (44.2)	1152 (32.7)
Body mass index				
Obese	1600 (21.4)	193 (12.1)	678 (42.4)	729 (45.6)
Overweight	2815 (37.7)	483 (17.2)	1167 (41.5)	1165 (41.4)
Normal	3061 (40.9)	917 (30.0)	1198 (39.1)	946 (30.9)
Comorbidity (diabetes)				
Yes	936 (12.3)	125 (13.4)	346 (37)	465 (49.7)
No	6648 (87.7)	1492 (22.4)	2739 (41.2)	2417 (36.4)
Family history (hypertension)				
Yes	2589 (34.3)	517 (20.0)	1117 (43.1)	955 (36.9)
No	4965 (65.7)	1096 (22.1)	1955 (39.4)	1914 (38.5)
Consume fruits (≥1 serving/day)				
No	3132 (41.3)	636 (20.3)	1242 (39.7)	1254 (40.0)
Yes	4453 (58.7)	982 (22.1)	1843 (41.4)	1628 (36.6)

**Table 2:** Multinomial Logistic Regression of Factors Associated with Prehypertension and Hypertension (*N* = 7585)

Variables	Prehypertension			Hypertension		
	Β	AOR (95% CI)	*P*-value	β	AOR (95% CI)	*P*-value
Age (year)						
<50						
≥50	0.544	1.723 (1.494-1.987)	<0.001*	1.413	4.108 (3.528-4.782)	<0.001*
Gender						
Male	0.534	1.706 (1.478-1.969)	<0.001*	0.697	2.008 (1.725-2.338)	<0.001*
Female						
Education level						
Low	–0.024	0.976 (0.834-1.142)	0.762	0.299	1.349 (1.146-1.588)	<0.001*
High						
Location						
Rural	0.527	1.694 (1.475-1.946)	<0.001*	0.651	1.918 (1.653-2.225)	<0.001*
Urban						
Employment status						
No	0.044	1.045 (0.905-1.205)	0.549	0.300	1.35 (1.16-1.572)	<0.001*
Yes						
Body mass index						
Obese	1.116	3.052 (2.53-3.68)	<0.001*	1.527	4.606 (3.784-5.606)	<0.001*
Overweight	0.694	2.002 (1.739-2.305)	<0.001*	0.998	2.713 (2.333-3.155)	<0.001*
Normal						
Comorbidity (diabetes)						
Yes	0.188	1.206 (0.964-1.51)	0.101	0.388	1.474 (1.178-1.844)	0.001*
No						
Family history (hypertension)						
Yes	0.330	1.391 (1.21-1.598)	<0.001*	0.441	1.555 (1.339-1.804)	<0.001*
No						
Consume fruits (≥1 serving/day)						
No	0.065	1.067 (0.938-1.213)	0.325	0.226	1.253 (1.094-1.436)	0.001*
Yes						

*Significant at *P*-value < 0.05, R^2^ = 17.4%.

Participants who were 50 years of age and older had 4 times the odds of having hypertension (OR: 4.108; 95% CI: 3.528, 4.782) than participants below 50 years of age. Males (OR: 2.008; 95% CI: 1.725, 2.338) and those who resided in rural areas (OR: 1.349; 95% CI: 1.146, 1.588) were nearly twice as likely to have hypertension as females and urban residents. Respondents with hypertension also more frequently reported having a lower education level (OR: 1.918; 95% CI: 1.653, 2.225) and being unemployed (OR: 1.350; 95% CI: 1.160, 1.572).

Respondents who were overweight and obese were nearly 3 times (OR: 2.713; 95% CI: 2.333, 3.155) and 5 times (OR: 4.606; 95% CI: 3.784, 5.606) more likely to have hypertension, respectively, than those with a normal BMI. Having diabetes (OR: 1.474; 95% CI: 1.178, 1.844) and a family history of hypertension (OR: 1.555; 95% CI: 1.339, 1.804) were also associated with an increased risk of hypertension. In terms of dietary intake, those with less than 1 serving of fruit per day were more likely to have hypertension (OR: 1.253; 95% CI: 1.094, 1.436) than those who consumed at least 1 serving of fruit per day.

## 4 Discussion

Data from the present study revealed that 40.7% and 38.0% of adults in this Malaysian cohort had prehypertension and hypertension, respectively. The prevalence of prehypertension was slightly higher in this study than the prevalence reported in the NHMS III, which was 37.0%,^17^ and a recent study by Rafan and colleagues, in which it was 37.1%.^18^ The prevalence of hypertension was also higher in this study than the prevalence reported in three consecutive NHMS reports in 2011 (32.7%), 2015 (30.3%), and 2019 (30.0%).^6^, ^7^, ^8^ The higher prevalence found in this study was expected, as the age range encompassed in the NHMS is wider, including those aged 7 years and above, while the present study included only adults between the ages of 35 and 70 years. In terms of population age, the age range utilized in this study is highly associated with risk factors for non-communicable diseases (NCDs), such as hypertension.

This study found that age was significantly associated with prehypertension and hypertension, with older adults showing higher odds. This is consistent with the NHMS 2019, in which the reported prevalence of hypertension was 9.8% in those between 30 and 39 years of age, while it was 65.0% in those 75 years of age and above.^8^ This has been explained in many studies as occurring due to the stiffening of blood vessels as humans age.^19^, ^20^, ^21^ This was further explained by Safar and Pinto,^22^, ^23^ who reported that changes in arterial and arteriolar stiffness may also be induced by high-salt diets due to the promotion of changes in vascular smooth muscle cells, resulting in collagen accumulation in the large artery walls and thus increased arterial stiffness.^22^^,^^23^ A local study including 761 adults suggested that for every 1-year increase in age, the odds of developing prehypertension increases by 1.06 times.^24^

Men were nearly 2 times more likely to have prehypertension and hypertension than women, which is supported by the findings of other studies conducted locally and in other countries.^18^^,^^25^^,^^26^ Nasir and colleagues^27^ found similar results in a large community study involving 11,288 adults aged ≥30 years, as males had 1.76 times the odds of having prehypertension than females.^27^ The reasons for gender differences in blood pressure are relatively unknown but a plausible explanation may be the protective effect of estrogen in women, which helps to regulate and protect against high blood pressure, particularly during the reproductive years.^28^, ^29^, ^30^ In addition, behavioral risk factors such as smoking, alcohol consumption, and tobacco use, which are highly associated with hypertension, are typically more common in men in the Asian population.^31^^,^^18^

The population residing in rural areas was more likely to have prehypertension and hypertension than those living in urban areas. This finding was in line with the NHMS report that rural populations had a prevalence of 17.2% while urban populations had a prevalence of 15.5%.^8^ The higher prevalence in the rural population has been said to be due to insufficient awareness among rural residents, particularly of the importance of early detection of NCDs, including hypertension.^32^, ^55^ A further analysis was done to calculate the awareness level of this study population by comparing the urban and rural areas. About 39% of those with hypertension in urban areas were diagnosed based on family history of hypertension as opposed to slightly lower population of 30.5% in rural areas (Table 3). A low doctor-to-population ratio of 1:682 in extremely rural areas such as Borneo may also hinder access to medical doctors, causing delays in treatment.^33^ An additional reason may relate to the tendency of the rural community to opt for traditional and complementary medicine to treat hypertension before seeking modern treatment.^9^^,^^34^^,^^35^ Late detection with low awareness of disease management and control may worsen hypertensive conditions, especially in those with prehypertension who are at risk of progressing to a hypertensive state.

**Table 3:** Hypertension Awareness Among Respondents with Hypertension by Location and Education Level

Hypertension (*N*)	Location	
	Rural	Urban
	1955	927
*Awareness	$\frac{596}{1955}\;\times \;100\%=\;30.5\%$	$\frac{359}{927}\;\times \;100\%=\;38.7\%$
	Education level	
	Low	High
	1600	1282
	$\frac{379}{1600}=\;23.7\%$	$\frac{576}{1282}=\;44.9\%$

⁎ Awareness = (respondents diagnosed with hypertension through family history/respondents diagnosed with hypertension) × 100%.

Epidemiological evidence strongly suggests a close relationship between increased BMI and hypertension.^36^^,^^37^ According to the Framingham Heart Study, the probability of developing hypertension is double in obese men and nearly triple in obese women compared to those with normal weight.^38^ A cross-sectional study performed on 1107 adults found elevated fatty tissue levels in obese individuals, which may increase vascular resistance and in turn increase the workload required for the heart to pump blood throughout the body.^39^ Another study stated that the additional blood flow required to perfuse the excess adipose tissue mass and the inability of the heart to cope with the increased cardiac output result in increased blood pressure.^40^

Having family members with hypertension was associated with a higher risk of prehypertension and hypertension compared to not having a family history. Those who grow up in the same household as a family member with hypertension may be exposed to high-risk behaviors, including dietary and physical activity habits. Individuals who are raised in a family that lacks awareness of healthy eating and physical activity may have a higher risk of developing prehypertension and hypertension compared to those raised in families practicing healthy lifestyles.^41^^,^^42^ In addition, studies have shown that genetic factors, such as high sodium–lithium countertransport, elevated uric acid levels, high fasting plasma insulin concentrations, fat pattern index, and BMI can contribute to the relationship between hypertension and family history.^43^, ^44^, ^45^

This study also revealed associated factors unique to hypertension, which included low education level, unemployment, and having comorbidity of diabetes. Low education level was often linked with low health literacy which may limit their ability to read and understand written educational materials (functional skills), communicate with healthcare professionals (interactive skills), and make appropriate health decisions (critical skills)^11^, ^12^, ^13^ A further analysis showed a lower awareness level of hypertension (diagnosed based from family history of hypertension) among those with low education level (24%) compared to those with higher education level (45%) (Table 3). They also may have fewer opportunities to be employed or, if any, may have jobs that come with lack of health benefits packages.^46^^,^^47^ Jobs with lower salary may result in difficulties in maintaining their financial status, which impacts stress levels, leading to poorer health and diseases such as hypertension.^48^^,^^49^ Moreover, they may have greater barriers to attaining timely healthcare, leading to delayed treatment and higher risk of hypertension-related complications.^50^^,^^46^

This study showed that individuals with diabetes were more likely to have hypertension than those without diabetes. Research has demonstrated a positive association between blood glucose abnormalities and blood pressure, with diabetes causing nearly 50% more risk of stage 1 hypertension.^51^, ^52^, ^56^ Evidence from the Strong Heart Study database of 4549 adults showed that the risk of developing more severe stages of hypertension was almost twofold higher in those with diabetes than those without.^47^ This has been explained by macroalbuminuria and microalbuminuria, which, according to a study by Wang et al.,^47^ occur primarily in individuals with diabetes, and/or microvascular damage due to chronic hyperglycemia.^47^

Fruit intake of at least 1 serving per day was shown to be a protective factor against hypertension in this study population. The mechanisms by which fruits may reduce the risk of hypertension are probably multiple. One hypothesis pertains to the high nutrients, phytochemicals, and flavonoid content in fruits. ^53^ In a randomized control trial (RCT) study shown a type of flavonoid found in apples (quercetin) was found to decrease SBP by 3 mmHg (*P* < .01) when compared with placebo in a double-blind crossover trial.^54^ Consuming fruit, which is a good source of fiber, limits the intake of other food, as fiber helps to maintain fullness. Although one serving of fruit per day does not meet the recommended fruit intake of two servings per day specified by the MOH, the findings of this study stress the importance of consuming fruit, as it may significantly reduce the risk of hypertension.

There are several limitations to our study. First, this study was a cross-sectional study in which the temporal link between the outcome and the exposure cannot be determined because both were examined at the same time. Second, the fruit intake among the respondents was assessed through FFQ which was based on respondents’ estimation and subject to recall bias. However, during the interview with respondents, the researchers brought along pictures of kitchen utensils used such as rice scoop and measurement of serving plate and bowl to ensure that the correct serving size was referred to by the respondents. Apart from that, this study managed to involve more than 7000 participants covering the entirety of Malaysia.

## 5 Conclusion

This study indicates the specific group that should be prioritized by health practitioners in awareness and early detection programs in Malaysia, which are those who are: 50 years old and above, males, residing in rural areas, overweight and obese, and having at least 1 family member with hypertension. Since this study has revealed that both overweight and obese were risk factors associated with prehypertension and hypertension, implementing simple lifestyle modifications involving tailored prescription of healthier balanced diets and effective physical activity, which aid in achieving and maintaining optimum BMI, should be the way forward to prevent and manage prehypertension and hypertension. Furthermore, early detection, particularly in those with prehypertension, will optimize the management of the disease.

## Authors’ Contributions

Conceptualization, N.H.I (Noor Hassim Ismail), R.I (Rosnah Ismail), A.M.T (Azmi Mohd Tamil), M.H.J (Mohd Hasni Ja'afar), and Z.M.I (Zaleha Md Isa); data collection, research assistants from HUKM and UiTM; data analysis, N.Z.A (Najihah Zainol Abidin), N.H.A.R (Nurul Hafiza Ab Razak), and K.H.Y (Khairul Hazdi Yusuf); funding acquisition, N.H.I, R.I, and M.H.J; methodology, Z.M.I, N.H.I, R.I, A.M.T, M.H.J, and K.H.Y; writing—original draft, N.Z.A and R.I; and writing—review and editing, N.Z.A, Z.M.I, N.H.A.R, N.H.I, R.I, A.M.T, M.H.J, N.M.N (Nafiza Mat Nasir), S.A.R (Suraya Abdul-Razak), and P.J (Philip Joseph). All authors have read and agreed to the published version of the manuscript.

## Funding

This work was supported by funded by the Population Health Research Institute, the Canadian Institutes of Health Research; Heart and Stroke Foundation of Ontario; and unrestricted grants from several pharmaceutical companies (grant no.: 101414). It was also supported by local grants from the Ministry of Science, Technology and Innovation of Malaysia (grant Nbr 100 - IRDC/BIOTEK 16/6/21 (13/2007), grant no.: 07-05-IFN-BPH 010), Ministry of Higher Education of Malaysia (grant Nbr 600 - RMI/LRGS/5/3 (2/2011)), UiTM and UKM (PHUM-2012-01).

## Role of Funding Source

The funders of the study had no role in its design, data collection, data analysis, data interpretation, writing of the report, or the decision to submit the paper for publication. The international and local funders only provided financial support without any intervention at any stage of the study. However, the outcomes of the study once published need to be reported to them for the purpose of grant output progress. The lead and senior authors (N.H.I. and R.I.) had full access to the Malaysian PURE data in the study and all authors had the final responsibility for the decision to submit for publication.

## Data Availability

The data underlying this article will be shared on reasonable request to the corresponding author.

## Declaration of Competing Interest

The authors declare that they have no known competing financial interests or personal relationships that could have appeared to influence the work reported in this paper.
